# Dietary iron intake and breast cancer risk: modulation by an antioxidant supplementation

**DOI:** 10.18632/oncotarget.12592

**Published:** 2016-10-12

**Authors:** Abou Diallo, Mélanie Deschasaux, Valentin Partula, Paule Latino-Martel, Bernard Srour, Serge Hercberg, Pilar Galan, Philippine Fassier, Françoise Guéraud, Fabrice H. Pierre, Mathilde Touvier

**Affiliations:** ^1^ Sorbonne Paris Cité Epidemiology and Statistics Research Center (CRESS), Inserm U1153, Inra U1125, Cnam, Paris 5, 7 and 13 Universities, Nutritional Epidemiology Research Team (EREN), Bobigny, France; ^2^ Public Health Department, Avicenne Hospital, Bobigny, France; ^3^ French Network for Nutrition And Cancer Research (NACRe Network), Bobigny, France; ^4^ UMR 1331 Toxalim, INRA/INP/UPS, Toulouse, France

**Keywords:** breast cancer, dietary iron, antioxidants, lipid peroxidation, prospective study

## Abstract

Experimental results suggested that iron-induced lipid peroxidation may explain the direct associations observed between red/processed meat intakes and colorectal and breast cancer risk. However, epidemiological evidence is lacking. Thus, we investigated the association between dietary iron intake and breast cancer risk, and its potential modulation by an antioxidant supplementation and lipid intake. This prospective study included 4646 women from the SU.VI.MAX trial (daily low-dose antioxidants vs. placebo). 188 incident breast cancers were diagnosed (median follow-up=12.6y). Dietary iron intake was assessed using repeated 24h dietary records. Multivariable Cox proportional hazards models were computed. Dietary iron intake was associated with an increased breast cancer risk (HR_T3vs.T1_=1.67 (1.02-2.71), P-trend=0.04). This association was observed in the placebo group (HR_T3vs.T1_=2.80 (1.42-5.54), P-trend=0.003), but not in the antioxidant-supplemented group (P-trend=0.7, P-interaction=0.1). Besides, in the placebo group, the increased breast cancer risk associated with dietary iron intake was more specifically observed in women with higher lipid intake (P-trend=0.046). These findings suggest that dietary iron intake may be associated with an increased breast cancer risk, especially in women who did not received antioxidants during the trial and who consumed more lipids. This supports the experimental results suggesting that breast cancer risk may be increased by iron-induced lipid peroxidation.

## INTRODUCTION

Recently, the International Agency for Research on Cancer (IARC) classified red and processed meat consumption as “(probably) carcinogenic to humans” (Group 2A and 1 respectively) [1]. Although these conclusions were mainly based on colorectal cancer risk [1, 2], existing evidence also suggests a positive association with other cancer sites such as female breast [3–6]. Notably, we previously observed an increased breast cancer risk associated with processed meat intake in the Supplémentation en Vitamines et Minéraux Antioxydants (SU.VI.MAX) cohort [6].

These associations could be explained by several potential pro-carcinogenic compounds found in red and processed meat such as heme iron, heterocyclic amines or *N*-nitroso compounds [1]. With an experimental approach on rodent models, we recently demonstrated that among all these potential pro-carcinogens, iron, as a pro-oxidant, may be of particular importance in the promotion of colon carcinogenesis [7, 8]. Furthermore, a possible role of elevated iron intake in breast carcinogenesis has been hypothesized [9, 10], in particular through its involvement in lipid peroxidation. Indeed, the interaction between lipids and iron in the intestinal tract may form lipid peroxidation end-products that are able to reach the systemic blood circulation and to induce oxidative stress in other sites [11, 12].

Epidemiological evidence regarding the association between iron intake and breast cancer risk is still limited and did not allow the World Cancer Research Fund/American Institute for Cancer Research (WCRF/AICR) to draw any conclusion [13]. To our knowledge, only five prospective studies are available [14–18]. Three of them observed null results [14, 16, 18] while the other two observed a direct association between iron intake and breast cancer risk in post-menopausal women [15, 17].

Since iron may promote breast carcinogenesis in particular through lipid peroxidation, it could be hypothesized that iron intake may all the more increase cancer risk as diet has a low antioxidant potential and high lipid content.

To our knowledge, no epidemiological study has investigated a potential modification of the association between iron intake and breast cancer risk by antioxidant or lipid intakes. In a previous work from the SU.VI.MAX cohort [6], the positive association between processed meat intake and breast cancer risk observed in the overall population was no longer observed when analyses were restricted to women who received an antioxidant supplementation. This work suggested that antioxidant intake may counteract some of the deleterious effect of processed meat intake on breast carcinogenesis, such as lipid peroxidation induced by iron.

Thus, our objectives were to prospectively investigate the association between dietary iron intake and breast cancer risk, and to study whether this association was modified by the antioxidant supplementation of the SU.VI.MAX trial and by lipid intake.

## RESULTS

During a median follow-up of 12.6y (52,500 person-years), 188 women developed a first primary breast cancer with a mean age at diagnosis of 55.7±7.0y.

Characteristics of participants according to tertiles of total dietary iron intake are presented in Table 1. Participants in the upper tertile tended to have a higher educational level, to be less physically active, and to have higher intakes of energy, alcohol and lipids. Mean ± SD of total dietary iron intake per subject was 10.9±3.2 g/d. Overall, women who provided at least three 24h dietary records (compared to women who did not) were slightly smaller and thinner, were more likely to take hormonal treatment for menopause and to have a family history of breast cancer and were less likely to drink alcohol or to smoke [data not tabulated].

**Table 1:** Baseline characteristics of participants according to tertiles of iron intake, SU.VI.MAX cohort, france, 1994–2007^a^

	Tertile 1 (n=1548)	Tertile 2 (n=1549)	Tertile 3 (n=1549)	P^b^
Age, y	47.2 ± 6.6	46.7 ± 6.6	47.1 ± 6.5	0.9
Children, n	2.0 ± 1.1	1.9 ± 1.2	1.9 ± 1.1	0.2
Height, cm	160.9 ± 5.7	161.9 ± 5.9	162.7 ± 5.9	0.3
Body mass index, kg/m^2^	23.2 ± 3.8	23.0 ± 3.7	23.1 ± 3.8	0.8
Menopause, yes	486 (31.4)	436 (28.1)	475 (30.7)	0.1
Hormonal treatment for menopause, yes	458 (29.6)	436 (28.1)	479 (30.9)	0.2
Intervention group of the initial trial				0.4
Antioxidant supplementation group	751 (48.5)	765 (49.4)	788 (50.9)	
Placebo group	797 (51.5)	784 (50.6)	761 (49.1)	
Family history of breast cancer^c^, yes	146 (9.4)	135 (8.7)	124 (8.0)	0.4
Smoking status				0.07
Never	920 (59.4)	891 (57.5)	868 (56.0)	
Former	406 (26.2)	462 (29.8)	472 (30.5)	
Current	222 (14.3)	196 (12.7)	209 (13.5)	
Physical activity				0.02
Irregular	419 (27.1)	377 (24.3)	394 (25.4)	
< 1 h/d walking or equivalent	489 (31.6)	563 (36.3)	569 (36.7)	
≥ 1 h/d walking or equivalent	640 (41.3)	609 (39.3)	586 (37.8)	
Educational level				<.0001
Primary	353 (22.8)	263 (17.0)	231 (14.9)	
Secondary	609 (39.3)	628 (40.5)	595 (38.4)	
University	586 (37.9)	658 (42.5)	723 (46.7)	
Alcohol intake, g/d	5.2 ± 6.8	10.0 ± 10.9	17.0 ± 17.1	<.0001
Energy intake, kcal/d	1392 ± 312	1777 ± 313	2088 ± 424	<.0001
Dietary iron, mg/d	7.7 ± 1.3	10.6 ± 0.7	14.4 ± 2.3	<.0001
Total lipids, g/d	63.0 ± 17.0	80.4 ± 17.9	95.7 ± 23.6	<.0001
Eicosapentaenoic acid (EPA), g/d	0.1 ± 0.1	0.1 ± 0.1	0.1 ± 0.1	<.0001
Docosahexaenoic acid (DHA), g/d	0.2 ± 0.2	0.2 ± 0.2	0.2 ± 0.2	<.0001

SU.VI.MAX, Supplémentation en Vitamines et Minéraux Antioxydants.

a Values are means ± SDs or N (%). Cut-offs for tertiles of total dietary iron intake were 9.3 and 11.9 mg/d.

b P value for the comparison between tertiles of iron intake using χ^2^ tests or Fisher tests (P-trend) as appropriate. All statistical tests were 2-sided.

c Among first-degree relatives.

Table 2 displays the associations between tertiles of dietary iron intake and breast cancer risk overall and according to menopausal status. Higher iron intake was associated with an increased breast cancer risk overall (HR_T3vs.T1_=1.67 (1.02, 2.71), P-trend=0.04) and in post-menopausal women (HR_T3vs.T1_=1.85 (1.02, 3.34), P-trend=0.04). No association was detected in analyses restricted to pre-menopausal women (HR_T3vs.T1_=1.39 (0.58, 3.29), P-trend=0.4), but the number of cases was limited (59 cases/3190 non-cases). Similar results were observed for iron intake from processed meat (overall, HR_T3vs.T1_=1.60 (1.07, 2.37), P-trend=0.02) but not from red meat (overall, HR_T3vs.T1_=1.00 (0.70, 1.43), P-trend=0.9) [data not tabulated].

**Table 2:** Associations between tertiles of dietary iron intake and breast cancer risk from multivariable cox proportional hazards models, SU.VI.MAX cohort, france, 1994–2007^a^, ^b^

	N for cases/non-cases	HR (95% CI)	P-trend
All women			0.04
Tertile 1	53/1495	1.00	
Tertile 2	57/1492	1.18 (0.78, 1.79)	
Tertile 3	78/1471	1.67 (1.02, 2.71)	
Premenopausal women			0.4
Tertile 1	17/1045	1.00	
Tertile 2	19/1094	1.05 (0.51, 2.18)	
Tertile 3	23/1051	1.39 (0.58, 3.29)	
Postmenopausal women			0.04
Tertile 1	36/1143	1.00	
Tertile 2	38/1093	1.25 (0.75, 2.08)	
Tertile 3	55/1107	1.85 (1.02, 3.34)	

CI, confidence interval, HR, Hazard ratio, SU.VI.MAX, Supplémentation en Vitamines et Minéraux Antioxydants

a Multivariable models were adjusted for age (timescale), energy intake without alcohol, intervention group of the initial SU.VI.MAX trial, number of 24-h dietary records, smoking status, educational level, physical activity, height, BMI, alcohol intake, family history of breast cancer, lipid intake, use of hormone replacement therapy, number of children and for premenopausal women: use of contraceptive pill, heavy period, and use of a hormonal intrauterine system.

b Cut-offs for tertiles of dietary iron intake were 9.3 and 11.9 mg/d.

The association between total dietary iron intake and breast cancer risk was modulated by antioxidant intake (Table 3, P-interaction=0.1): in stratified analyses according to the intervention group of the SU.VI.MAX trial, higher iron intakes were associated with an increased breast cancer risk in the placebo group (HR_T3vs.T1_=2.80 (1.42, 5.54), P-trend=0.003) but not in the group supplemented with antioxidants (HR_T3vs.T1_=0.86 (0.43, 1.74), P-trend=0.7). A similar modulation was observed when analyses were restricted to post-menopausal women (placebo group: P-trend=0.03, supplemented group: P-trend=0.6, P-interaction=0.6) or to pre-menopausal women (placebo group: P-trend=0.02, supplemented group: P-trend=0.2, P-interaction=0.04).

**Table 3:** Associations between tertiles of dietary iron intake and breast cancer risk from multivariable cox proportional hazards models, stratified by antioxidant/placebo group of the SU.VI.MAX trial, france, 1994–2007^a^, ^b^

	Placebo group			Antioxidant supplementation group			
	N for cases/non- cases	HR (95% CI)	P-trend	N for cases/non- cases	HR (95% CI)	P-trend	P-interaction^c^
All women			0.003			0.7	0.1
Tertile 1	25/772	1.00		28/723	1.00		
Tertile 2	33/751	1.83 (1.03, 3.25)		24/741	0.70 (0.38, 1.29)		
Tertile 3	42/719	2.80 (1.42, 5.54)		36/752	0.86 (0.43, 1.74)		
Premenopausal women			0.02			0.2	0.04
Tertile 1	7/545	1.00		10/500	1.00		
Tertile 2	10/549	1.83 (0.62, 5.39)		9/545	0.60 (0.21, 1.68)		
Tertile 3	16/521	3.87 (1.16, 12.86)		7/530	0.39 (0.1, 1.56)		
Postmenopausal women			0.03			0.6	0.6
Tertile 1	18/593	1.00		18/550	1.00		
Tertile 2	23/544	1.90 (0.96, 3.76)		15/549	0.74 (0.35, 1.59)		
Tertile 3	26/535	2.49 (1.08, 5.74)		29/572	1.18 (0.51, 2.73)		

CI, confidence interval, HR, Hazard ratio, SU.VI.MAX, Supplémentation en Vitamines et Minéraux Antioxydants

a Multivariable models were adjusted for age (timescale), energy intake without alcohol, intervention group of the initial SU.VI.MAX trial, number of 24-h dietary records, smoking status, educational level, physical activity, height, BMI, alcohol intake, family history of breast cancer, lipid intake, use of hormone replacement therapy, number of children and for premenopausal women: use of contraceptive pill, heavy period, and use of a hormonal intrauterine system.

b Cut-offs for tertiles of dietary iron intake were 9.3 and 11.9 mg/d.

c Between dietary iron intake and supplementation group

A further exploratory stratification was performed according to the median intake of total lipids (Table 4). No association was observed in the group supplemented with antioxidants, whatever the level of lipid intake. In the placebo group, although P for interaction was not statistically significant (P-interaction=0.3), different associations were observed according to lipid intake: higher dietary intakes of total iron were positively associated with breast cancer risk in women with higher intakes of total lipids (≥ median, HR_T3vs.T1_=2.57 (0.86, 7.69), P-trend=0.046) while no significant association was detected in women with lower lipid intakes (< median, HR_T3vs.T1_=1.99 (0.79, 4.99), P-trend=0.1). Similar results were observed with two major long chain n-3 polyunsaturated fatty acids, docosahexaenoic acid (DHA) and eicosapentaenoic acid (EPA): no association in the antioxidant group, while in the placebo group, positive associations were observed in women with higher intakes (≥ median) of EPA (HR_T3vs.T1_=4.38 (1.58, 12.1), P-trend=0.004) and DHA (HR_T3vs.T1_=3.67 (1.30, 10.33), P-trend=0.01), but not in women with intakes < median (HR_T3vs.T1_= 1.77 (0.66, 4.72), P-trend=0.3 for EPA, and HR_T3vs.T1_= 2.15 (0.82, 5.66), P-trend=0.1 for DHA) (P-interactions=0.04 for EPA and 0.3 for DHA) [data not tabulated].

**Table 4:** Associations between tertiles of dietary iron intake and breast cancer risk from multivariable cox proportional hazards models, stratified by antioxidant/placebo group of the SU.VI.MAX trial and by lipid intake,france, 1994–@2007^a^, ^b^, ^c^

	Placebo group			Antioxidant supplementation group		
	N for cases/non-cases	HR (95% CI)	P-trend	N for cases/non-cases	HR (95% CI)	P-trend
**Total lipid intake < median (78.5 g/d)**			0.1			0.3
Tertile 1	20/632	1.00		21/602	1.00	
Tertile 2	18/338	1.58 (0.76, 3.24)		12/345	0.94 (0.41, 2.15)	
Tertile 3	12/143	1.99 (0.79, 4.99)		11/169	1.67 (0.63, 4.42)	
**Total lipid intake ≥ median (78.5 g/d)**			0.046			0.2
Tertile 1	5/140	1.00		7/121	1.00	
Tertile 2	15/413	1.41 (0.49, 4.06)		12/396	0.40 (0.15, 1.05)	
Tertile 3	30/576	2.57 (0.86, 7.68)		25/583	0.42 (0.15, 1.17)	

CI, confidence interval, HR, Hazard ratio, SU.VI.MAX, Supplémentation en Vitamines et Minéraux Antioxydants

a Multivariable models were adjusted for age (timescale), energy intake without alcohol, intervention group of the initial SU.VI.MAX trial, number of 24-h dietary records, smoking status, educational level, physical activity, height, BMI, alcohol intake, family history of breast cancer, lipids intake, use of hormone replacement therapy, and number of children.

^b^ Cut-offs for tertiles of dietary iron intake were 9.3 and 11.9 mg/d.

^c^ P for interaction between dietary iron and lipid intakes: Placebo group, 0.3; Antioxidant supplementation group, 0.5.

Results were similar when analyses were restricted to women who provided at least six (156 cases/3586 non-cases) or nine (116 cases/2737 non-cases) 24h-dietary records within the first two years of follow-up, when cases diagnosed within the first two years of follow-up were excluded (163 cases/4458 non-cases) or when *in situ* breast cancers were excluded (165 cases/4458 non-cases) [data not shown].

## DISCUSSION

In this prospective study, total dietary iron intake was associated with an increased risk of breast cancer. This association was no longer observed in the group supplemented with antioxidants during the SU.VI.MAX trial. In contrast, in the placebo group, a direct association was observed between total dietary iron intake and breast cancer risk, especially in women with higher intakes of total lipids (thus, with more precursors for lipid peroxidation), and notably EPA and DHA, two long chain n-3 polyunsatured fatty acids particularly prone to peroxidation because of their high number of double bonds [19].

To our knowledge, only five prospective studies were performed regarding the association between iron intake and breast cancer risk, with inconsistent results [14–18]. While three of them observed null results [14, 16, 18], our results are in line with those of two large prospective studies that observed a direct association between iron intake and postmenopausal breast cancer risk [15, 17]. Ferrucci et al. [17] observed a direct association with dietary iron intake in the Prostate, Lung, Colorectal, and Ovarian Cancer Screening Trial, but no association was observed with total iron (dietary + supplemental). Inoue-Choi et al. [15] observed a direct association with heme iron intake in the NIH-AARP Diet and Health Study. Some prospective epidemiological studies also reported a positive association between elevated blood iron concentration and breast cancer risk [20, 21].

Our results are consistent with the hypothesis that iron intake would increase breast cancer risk, through lipid peroxidation. To our knowledge, this epidemiological study was the first to investigate a potential modulation of the association between dietary iron intake and breast cancer risk by an antioxidant supplementation and by lipid intake. However, in a previous study performed in the SU.VI.MAX cohort [6], a direct association was observed between processed meat intake (rich in heme iron) and breast cancer risk, and this association was no longer significant in the group supplemented with antioxidants. No association was detected for red meat, probably because intakes were relatively low (below 500g/week) for most women in this study [6]. These results suggested that the antioxidants may have counteracted some of the deleterious effects of dietary iron towards breast cancer either by preventing the lipid peroxidation or by protecting the cells against the oxidative stress induced by lipid peroxidation end-products. In this way, in a recent publication on the E3N cohort, we have observed that the positive association between heme iron intake and risk of colorectal adenoma was only observed in women with a total dietary antioxidant capacity ratio below the median cohort value [22]. These findings are in line with experimental data. Iron is considered as one of the major compounds explaining the association between red/processed meat intake and colorectal cancer risk [8]. Indeed, iron is a pro-oxidant involved in the production of reactive oxygen species that interact with all classes of macromolecules (e.g. DNA, lipid) [9]. In particular, the peroxidation of lipids leads to the formation of end-products involved in oxidative stress/damages and chronic inflammation [7, 9–11, 23]. Although these compounds may be mainly produced in the digestive tract from the interaction between oxidative compounds and lipids, they also appeared to be able to enter the systemic circulation [12] and thus to reach other organs. In particular, evidence from a case-control study reported that blood concentration of these end-products was increased in breast cancer patients [24]. Lipid peroxidation may thus be harmful to human health and could be involved in carcinogenesis, not only at the digestive tract level but also in non-digestive organs such as the breast [9, 10, 25].

Besides, experimental studies showed a protective effect of antioxidants, in particular vitamins C and E (both included in the SU.VI.MAX capsule), towards lipid peroxidation [26–28]: Vulcain et al. observed *in vitro* an inhibition of iron-induced lipid peroxidation by α-tocopherol [26]; Pierre et al. observed that when α-tocopherol was ingested in addition to cured meat, as compared to cured meat alone, there was a decrease in lipid peroxidation biomarkers in human volunteers [27]; and Klouche et al. showed that vitamins C and E inhibited the heme-induced oxidation of low-density lipoproteins [28]. Therefore, our results support the mechanistic hypotheses linking dietary iron and breast cancer through lipid peroxidation.

Strengths of our study include its prospective design with a long follow-up. The randomized control trial design of the SU.VI.MAX study allowed us to test the potential modification effect of an antioxidant supplementation at nutritional doses on the association between dietary iron and breast cancer. Dietary intakes were assessed by repeated 24-hour dietary records (mean of 9 records per subject) accounting for intra-individual variability (day-to-day and seasonal variations). The results were highly consistent with our initial hypotheses based on mechanistic data and were original regarding existing literature. Finally, a large range of confounding factors has been taken into account, thus limiting potential bias.

However, some limitations should also be acknowledged. First, no information was available regarding heme iron so that it was not possible to specifically test its association with breast cancer risk. Heme iron is supposed to be one of the major compounds explaining the association between red and processed meat and cancer [8]. However, total dietary iron intake may also be relevant when studying breast carcinogenesis [9]. Besides, as a proxy for heme iron, we investigated dietary iron from processed meat and from red meat in secondary analyses. The results (iron from processed meat: positive association, from red meat: no association) were consistent with those of the previous SU.VI.MAX study on red and processed meat intake and breast cancer risk presented above [6]. Second, we could not perform analyses on the association between other potential carcinogens from red and processed meat (heterocyclic amines or *N*-nitroso compounds) and breast cancer risk since this information was not available. Third, although the number of cases was appropriate for the main analyses reported here, it was nonetheless a limit in stratified analyses so that the results of these stratified analyses should be interpreted with caution. Finally, in SU.VI.MAX, participants received a combination of antioxidants (ascorbic acid, vitamin E, β-carotene, selenium and zinc) so that it was not possible to isolate the potential specific effect of each compound in the studied interaction.

This prospective study suggests that total dietary iron intake may be associated with an increased risk of breast cancer. For the first time, these results also suggest that this association could be modified by a supplementation with nutritional doses of antioxidants and by lipid intake, thus supporting the hypotheses raised by experimental studies that iron may increase breast cancer risk, in particular through lipid peroxidation. Indeed, dietary iron intake was associated with an increased breast cancer risk in women not supplemented with antioxidants, suggesting that antioxidants may counteract some of the deleterious effects of dietary iron on breast carcinogenesis, and in women with higher lipid intakes (i.e. with more substrates for lipid peroxidation). Although our results regarding the association between dietary iron intake and breast cancer risk were consistent with two large prospective studies [15, 17], they have to be confirmed in future large cohorts, and especially the observed interaction between iron and antioxidant intakes. If these results are confirmed and if the causality of the associations can be established, this may lead to formulate explicit public health recommendations towards the limitation of the consumption of iron-rich foods such as red and processed meat (also containing other carcinogenic compounds [1]) while promoting the consumption of antioxidant-rich foods.

## MATERIALS AND METHODS

### Participants

The SU.VI.MAX study was at first designed as a randomized, double-blind, placebo-controlled primary prevention trial (clinicaltrials.gov NCT00272428) aiming to assess the effect of a daily supplementation with nutritional doses of antioxidants (120 mg ascorbic acid, 30 mg vitamin E, 6 mg β-carotene, 100 μg selenium, and 20 mg zinc) versus a placebo, on the incidence of cardiovascular diseases and cancers [29]. In 1994–1995, 13,017 individuals, among which 7876 women (35-60y), were enrolled for an 8y intervention study. Follow-up of health events lasted until September 30^th^, 2007. 5.2% of participants were lost to follow-up. As reported before [30], the antioxidant supplementation was not associated with breast cancer risk in this trial. A graphical presentation of the study design with data collection phases is available in SupplementalFigure S1.

### Compliance with ethical standards

The SU.VI.MAX study was conducted according to the Declaration of Helsinki guidelines and was approved by the Paris-Cochin Hospital Ethics Committee for Studies with Human Subjects (CCPPRB nos.706 and 2364, respectively) and the French National Commission for Computed Data and Individual Freedom (CNIL nos. 334641 and 907094, respectively). Written informed consent was obtained from all participants.

### Baseline data collection

#### Dietary data

Every 2 months during the trial phase (1994–2002), participants were invited to complete a 24-h dietary record via the Minitel Telematic Network, a French telephone-based terminal equivalent to an Internet prototype used widely at the beginning of the study. The records were randomly distributed between weeks and weekends and over seasons to take into account intra-individual variability. Participants assessed portion sizes with a validated picture booklet [31], and the amounts consumed from composite dishes were estimated using French recipes validated by food and nutrition professionals. The mean daily energy, alcohol, macro- and micronutrient intakes were estimated using a published French food composition table [32]. Participants were advised against taking any self-prescribed supplementation during the trial.

#### Other covariates

Baseline information about socio-demographics, smoking status, physical activity, and family history of breast cancer were collected by self-administered questionnaires. Anthropometric measures (height and weight) were obtained during a medical examination by the study nurses and physicians.

#### Case ascertainment

During the follow-up period, participants were invited to self-report health events (through a monthly questionnaire). Investigations were then conducted to obtain medical data from participants, physicians, and/or hospitals. All information was reviewed by an independent physician expert committee. All cancer cases were documented by a pathology report and were validated by histologic reports. The International Chronic Diseases Classification, 10th Revision, Clinical Modification [33] was used to classify the cancer cases. All first-incident primary breast cancers were considered as cases in this study.

#### Statistical analyses

From the 7876 women included in the SU.VI.MAX study, we excluded those who reported a cancer diagnosis before the start of the follow-up (N=120) and those with a chronic inflammatory disease that may impact iron metabolism (N=58, among which 28 rheumatoid arthritis, 8 ankylosing spondylitis, 6 hemorrhagic rectocolitis, 5 hemochromatosis, and 11 others). Among the remaining participants, 4646 provided at least 3 valid 24-h dietary records within the first 2 years of follow-up and thus were included in the analyses (see the flowchart in SupplementalFigure S2). Food and nutrient intakes were assessed using mean intakes calculated from all dietary records provided during the first two years of follow-up for each woman.

Baseline characteristics of participants were compared between tertiles of total dietary iron intake using χ^2^ tests or Fisher tests (from ANOVA models) wherever appropriate. Hazard ratios (HR) and 95% confidence intervals (CI) obtained from Cox proportional hazards models, with age as the primary time variable, were used to characterize the association between tertiles of total dietary iron intake and the incidence of breast cancer. Participants contributed person-time until the date of diagnosis of breast cancer, the date of last completed questionnaire, the date of death, or September 30^th^, 2007, whichever occurred first. Participants who reported a cancer other than breast cancer during the study period were included and censored at the date of diagnosis (except those with basal cell skin carcinoma, which was not considered as cancer). We confirmed that the assumptions of proportionality were satisfied through examination of the log-log (survival) vs. log-time plots. Tests for linear trend were performed using the ordinal score on tertiles of total dietary iron intake.

Stratified analyses were performed according to menopausal status (premenopausal and postmenopausal). For these analyses, women contributed person-time until their date of menopause for premenopausal breast cancer analysis or from their date of menopause for postmenopausal breast cancer analysis.

Multivariable models were adjusted for factors constitutive to the study design [initial SU.VI.MAX trial intervention group (antioxidant/placebo), number of dietary records (continuous)], socio-demographic variables [age (time-scale) and educational level (primary, secondary, or university)], lifestyle factors [smoking status (never, former, or current), physical activity (irregular, <1h/d or ≥1h/d walking or equivalent), and alcohol intake (continuous)], anthropometric factors [height (continuous) and BMI (continuous)], dietary factors [dietary intakes of energy without alcohol (continuous), lipid intake (continuous)] and factors indicating higher individual susceptibility to breast cancer [family history of breast cancer (yes/no), menopausal status at baseline (yes/no), use of hormonal treatment for menopause at baseline (yes/no), number of children (continuous) and, for analyses restricted to premenopausal women, contraceptive pill (yes/no), heavy period (yes/no) and hormonal intrauterine system (yes/no)]. Interactions were tested between tertiles of dietary iron intake and 1) the antioxidant supplementation of the initial SU.VI.MAX trial and 2) lipid intake. Stratified analyses were performed according to the intervention group of the initial SU.VI.MAX trial (antioxidant/placebo) and further exploratory stratified analyses were also performed according to the median intakes of lipid.

For all covariates, less than 5% of values were missing and were replaced by the respective mode value.

Since we showed in a previous work [6] that processed meat intake was associated with an increased breast cancer risk (while no association was observed for red meat intake), we performed secondary analyses on the associations between dietary iron from red and from processed meat and breast cancer risk.

Sensitivity analyses were carried out by excluding women who provided less than six or nine 24h-dietary records within the first two years of follow-up, cases diagnosed within the first two years of follow-up, or *in situ* breast cancers.

All tests were two-sided, and P<0.05 was considered statistically significant. SAS version 9.3 (SAS Institute Inc., Cary, NC.) was used for the analyses.

## SUPPLEMENTARY FIGURES AND TABLES


