# Supplementary material for: Dietary iron intake and breast cancer risk: modulation by an antioxidant supplementation
Source: Oncotarget. 2016 Oct 12;7(48):79008–16. doi: 10.18632/oncotarget.12592 (PMC5346694; doi:10.18632/oncotarget.12592)
Supplement: Supplementary file 1 [file oncotarget-07-79008-s001.pdf]

Dietary iron intake and breast cancer risk: modulation by an antioxidant supplementation

SUPPLEMENTARY FIGURES

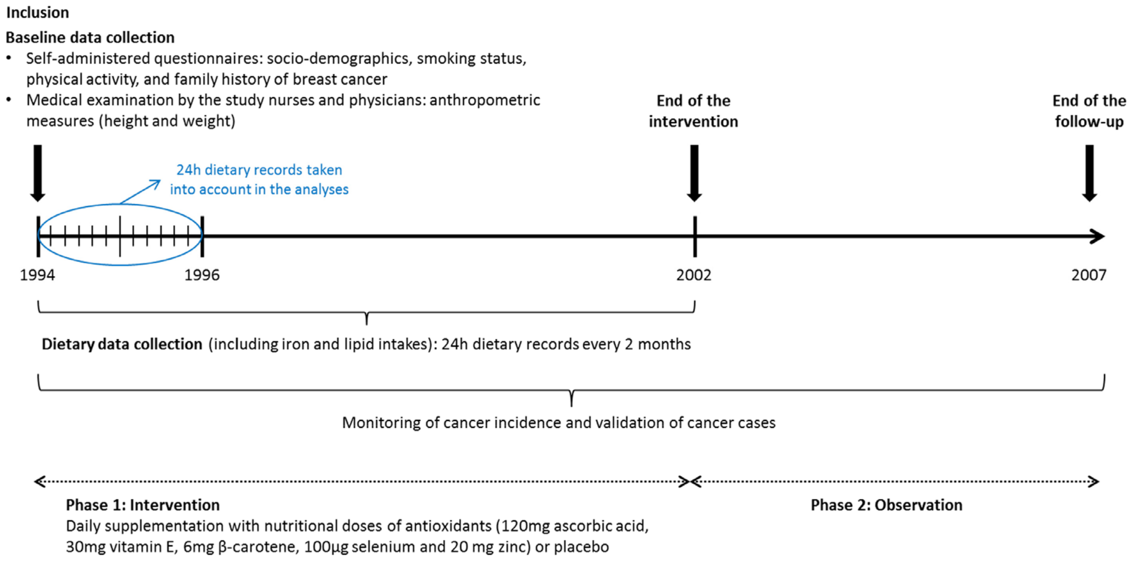

Supplementary Figure S1: Graphical presentation of the study design with data collection phases.

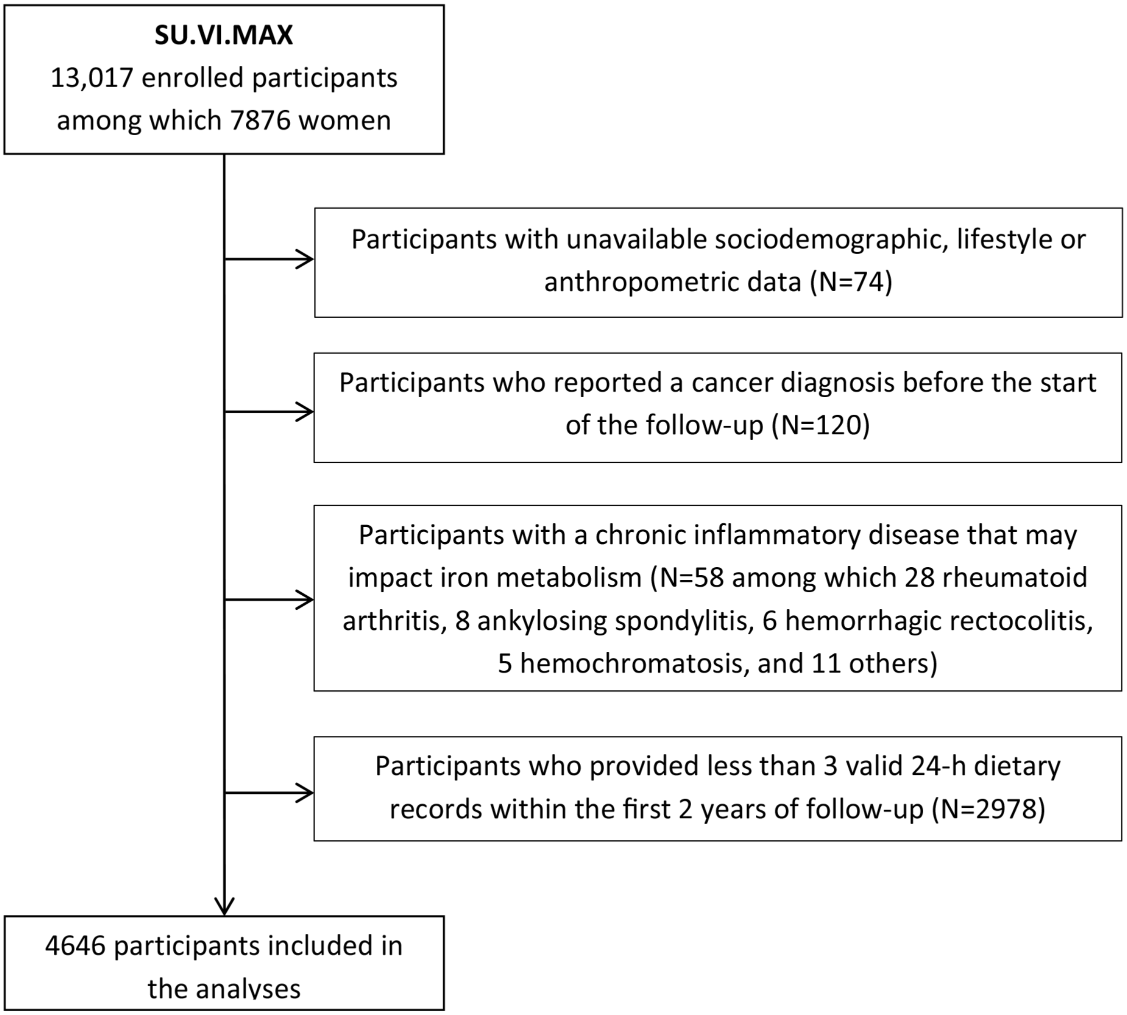

Supplementary Figure S2: Participants' flowchart.
